# The Role of Aryl Hydrocarbon Receptor and Crosstalk with Estrogen Receptor in Response of Breast Cancer Cells to the Novel Antitumor Agents Benzothiazoles and Aminoflavone

**DOI:** 10.4061/2011/923250

**Published:** 2011-09-22

**Authors:** Mariana A. Callero, Andrea I. Loaiza-Pérez

**Affiliations:** Research Area, Institute of Oncology “Ángel H. Roffo”, University of Buenos Aires, Avenue San Martín 5481, C1417DTB Ciudad de Buenos Aires, Argentina

## Abstract

Many estrogen-receptor- (ER-) expressing breast cancers become refractory to ER-based therapies. New antitumor drugs like aminoflavone (AF) and benzothiazoles (Bzs) have been developed and have exquisite antitumor activity in ER+MCF-7 and T47D cells and in a MCF-7 nude mouse model. ER(−) breast cancer cells like MDA-MB-231 are less susceptible. We previously found in MCF-7 cells that these drugs activate the aryl hydrocarbon receptor (AhR) via translocation to the nucleus, induction of AhR-specific DNA binding activity, and expression of CYP1A1, whose transcription is controlled by the AhR-ARNT transcription factor. CYP1A1 metabolizes AF and Bz to a species which directly or after further metabolism damages DNA. In contrast an AhR-deficient variant of MCF-7 or cells with predominantly nuclear AhR expression, such as MDA-MB 231, are resistant. Thus, these drugs, unlike other neoplastic agents, require AhR-mediated signaling to cause DNA damage. This is a new treatment strategy for breast cancers with intact AhR signaling.

## 1. Treatment Advances in Breast Cancer

Metastatic breast cancer is currently incurable, and novel strategies that might become useful treatments are needed. In the past decade, Herceptin directed against the HER2/neu oncoprotein and aromatase antagonists have entered clinical practice [1, 2]. Despite these advances, cytotoxicity evoked by drugs directed at DNA remains an interesting option [3]. However, these cytotoxics are nonspecific. Ideally, new breast cancer cytotoxics would engage some aspect of breast cancer biology to convey selective toxicity to breast cancer cells.

## 2. Aryl Hydrocarbon Receptor

The aryl hydrocarbon receptor (AhR) was initially defined as a receptor for environmental toxins such as dioxin. It belongs to the helix-loop-helix transcription factor family. Other members of this family are AhR nuclear translocator (ARNT); *Drosophila* proteins, SIM and PER, and hypoxia-inducible factor 1*α* (HIF 1*α*) [4–7]. AhR is a ligand-activated transcription factor. The most commonly known ligands of AhR are polycyclic and polyhalogenated hydrocarbons (benzopyrene, 3-methyl-colantrene), xenobiotics (phenobarbital), and other pesticides like tetrachlorodibenzo-p-dioxin (TCDD). 

**Figure 1:**
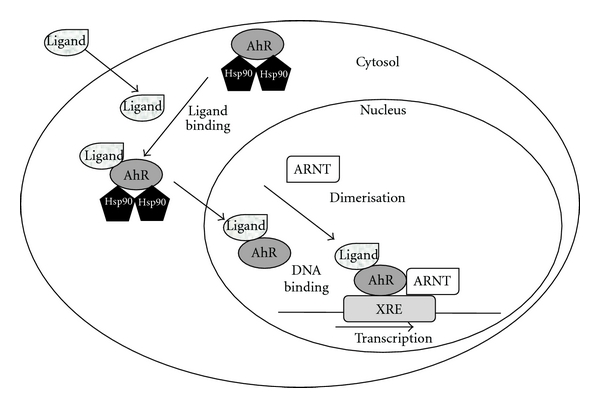
The aryl hydrocarbon signaling pathway.

AhR is localized within the cell cytosol constitutively where it is part of an inactivated complex composed of two heat-shock proteins: heat-shock protein 90 (Hsp90) and a 43 kDa protein known as AIP (Figure 1). The role of Hsp90 involves a chaperone activity that keeps AhR in a favorable ligand-binding configuration while it prevents its nuclear translocation. Hydrophobic ligands of AhR enter the cell by simple diffusion and bind to the receptor associated to Hsp90. Ligand binding to the receptor triggers a conformational change in AhR to a form that exhibits stronger affinity for DNA. This event leads to dissociation of the cytoplasmic complex and to AhR nuclear translocation. Within the nucleus, AhR interacts with ARNT forming a heterodimer that binds to specific DNA sequences called xenobiotic response elements (XREs). This binding leads to the transcriptional activation of genes that possess these XREs in their promoter sequences. Some of the genes activated by AhR encode phase I and II metabolic enzymes such as cytochrome P450 (CYP) 1A1, CYP1A2, and CYP1B1. AhR activation was first described as a cellular response to promote elimination of ambient contaminants and xenobiotics [8–10]. In humans, AhR is localized in liver, lungs, kidneys, placenta, lymphocytes, ovary, and breast. AhR/ARNT complex activation is tissue-specific and depends on co-regulators present in different cell types [9].

## 3. Estrogen Receptor-Aryl Hydrocarbon Receptor Crosstalk

It was demonstrated that in breast cancer cells, AhR ligands have the capacity to bind to ER and potentially interfere with ER signaling [11]. Also, it has long been known that estrogen can be metabolized by AhR-driven genes such as CYP1B1 to yield toxic metabolites that in some cases have been proposed to act as genotoxins [12]. This has led to the hypothesis that mutual modulation of AhR and ER signaling functions may be possible. Indeed, previous publications have shown that certain AhR ligands can have antiproliferative effects alone or in conjunction with ER antagonist administration with evidence of antitumor activity in breast cancer models [13]. How estrogen and its antagonist will agonize, have no effect, or amplify AhR-related signaling functions is a key unresolved question.

## 4. Benzothiazoles and Aminoflavone: AhR-Targeted Therapies for Breast Cancer

Empirical screening in the NCI cell line anticancer drug screen has revealed two types of molecules, the benzothiazoles (Bzs) [14–18] and aminoflavone (AF) [19], which are noteworthy for differential cytotoxicity. “Sensitive” cell lines have total growth inhibition (TGI) between 0.1 and 1 *μ*M, while “resistant” cell lines are refractory to Bz and AF concentrations <10 *μ*M. Among the consistently sensitive cell lines to both compound classes were the ER(+) breast cancer cell lines MCF-7 and T47D [18, 19]. While certain other cell types in this screen did show susceptibility, for example, renal cancer, in the breast cancer panel, optimal cytotoxicity of these drugs was seen in cell lines expressing estrogen receptor (ER(+)) [18]. Detailed mechanistic studies for both Bzs and AF have revealed that “sensitive” cells can activate AhR signaling, as might be expected from their planar nature [20]. This causes expression of CYP1A1 and in certain cell lines CYP1B1. Prior work had shown that CYP1A1 can metabolize Bzs and AF to produce DNA-damaging metabolites [14–19].

## 5. Benzothiazoles' Mechanism of Action

**Figure 2:**
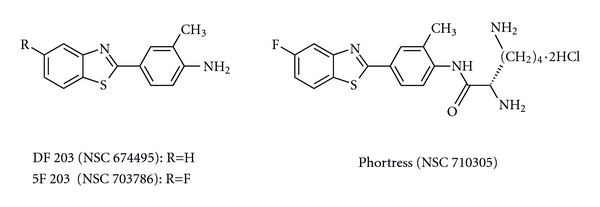
Chemical structures of antitumor 2-(4-amino-3-methylphenyl)benzothiazoles.

Previous results from our research group have demonstrated that the antitumor effect of compounds of the 2-(4-amino-3-methylphenyl) benzothiazole group (DF 203, NSC 674495; 5F 203, NSC 703786) (Figure 2) is mediated by AhR in MCF-7 breast tumor cells [15, 17, 21]. Currently Phortress, the lysine amide prodrug of 2-(4-amino-3-methylphenyl)-5-fluorobenzothiazole (5F 203), is under Phase I clinical evaluation sponsored by the Cancer Research UK [21–29].

**Figure 3:**
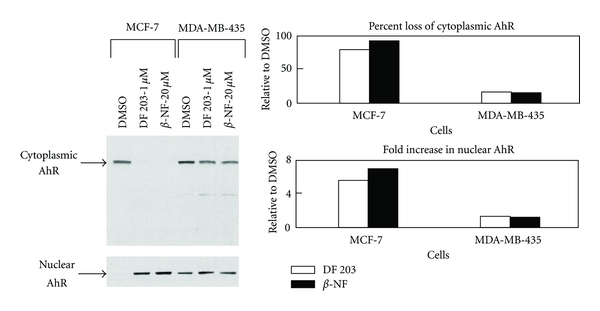
DF 203 causes an increase in immunoreactive nuclear AhR in MCF-7 cells. MCF-7 and MDA-MB-435 were treated with 0.1% DMSO, 1 *μ*M DF 203, or 20 *μ*M *β*-naphthoflavone for 1 h. Determination of immunoreactive AhR in cytoplasmic and nuclear fractions was performed by western blot. *β*-NF: *β*-naphthoflavone [15].

DF 203 preceded 5F 203 in the development of Phortress. A fluorine atom was introduced to thwart deactivating metabolism of DF 203 by CYP1A1 to inactive hydroxylated biotransformation products [23]. We observed that treatment of MCF-7 with Bzs resulted in activation of AhR.  Figure 3 shows AhR translocation to the nucleus after treatment with DF 203 in sensitive cells like MCF-7 but not in resistant cells like MDA-MB-435. However, a controversy exists concerning the origin of MDA-MB-435 cells. In the last years it has been shown that these cells are derived from a melanoma [15]. 

**Figure 4:**
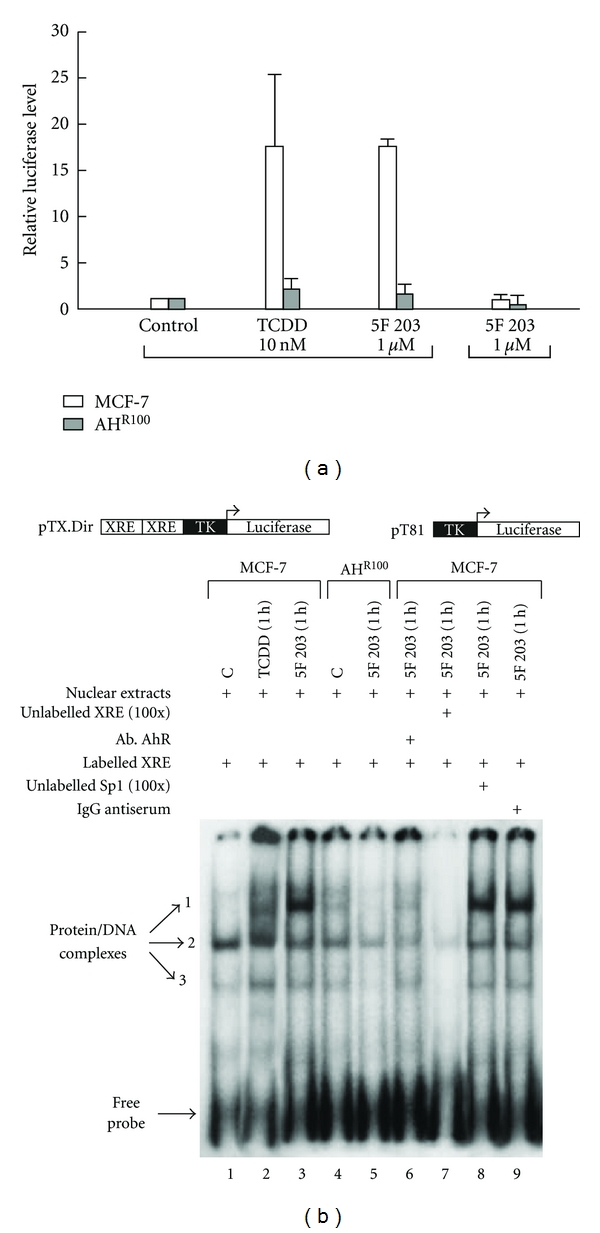
(a) 5F 203 induces binding to the XRE sequence of CYP1A1. MCF-7 and AHR100 cells were transfected with XRE-luciferase (pTX.Dir.) or pT81. A schematic of the respective construct is shown below the panel. Transfected cells were treated with DMSO, TCDD (10 nM), or 5F 203 (1 *μ*M) for 9 h. XRE-luciferase activity was determined normalizing to the amount of *Renilla reniformis* luciferase. The values are expressed as luciferase levels relative to control. (b) 5F 203 induces protein/DNA complexes on the XRE sequence of the CYP1A1 promoter. Nuclear extracts (20 mg) prepared from MCF-7 cells treated with 0.1% DMSO control (lane 1), TCDD (10 nM, 1 h) (lane 2), or 5F 203 (1 *μ*M, 1 h) (lane 3) were incubated with labeled XRE sequence derived from the CYP1A1 promoter for 10 min at room temperature. Free DNA and bound DNA were separated as described. In competition experiments, nuclear extracts from MCF-7 cells treated with 5F 203 (1 *μ*M, 1 h) were incubated with 4 *μ*g of anti-AhR antibody (lane 6), 100-fold excess of unlabeled XRE oligonucleotide (lane 7), 100-fold excess of unlabeled Sp1 oligonucleotide (lane 8), or 4 *μ*g of IgG antiserum (lane 9). Protein/DNA complexes from AHR100 cells were resolved in the same gel. Nuclear extracts from these cells (20 mg) treated with DMSO (lane 4) or 5F 203 (1 *μ*M, 1 h) (lane 5) were incubated with radioactive XRE and resolved by the same procedure [17].

In a similar way when we used 5F 203 we observed an increase in AhR transcriptional activity (increase in XRE-luciferase activity (Figure 4(a)) and formation of protein/DNA complexes bound to XRE Figure 4(b)) [17]. 

**Figure 5:**
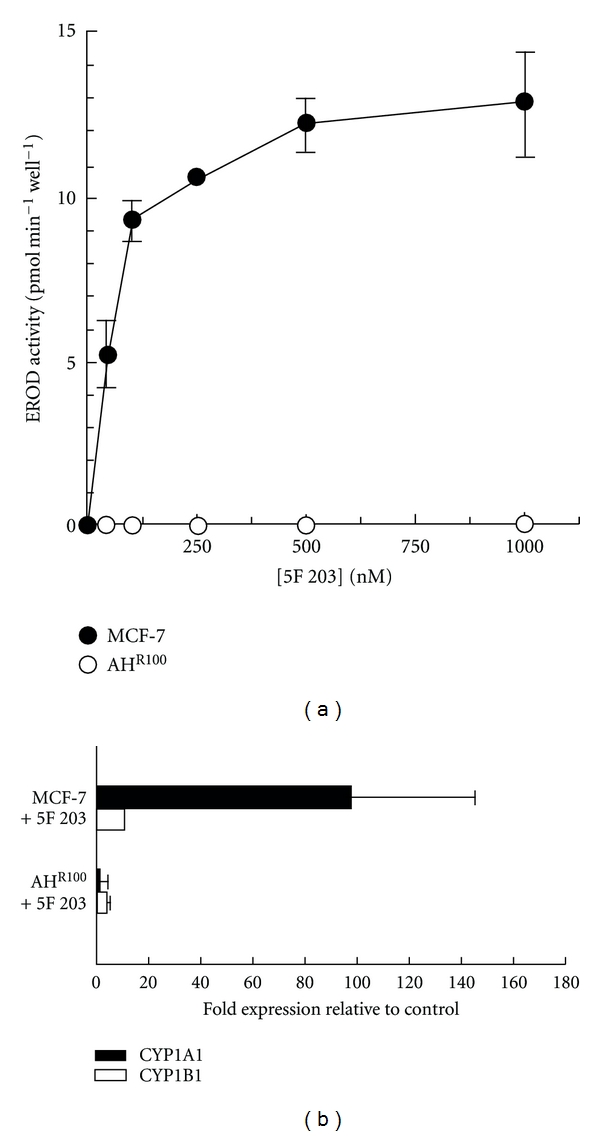
(a) 5F 203 induces CYP1A1 activity in MCF-7 but not in AH^R100^ cells. MCF-7 and AHR100 cells were incubated for 24 h with DMSO (0.1%) and 5F 203 (1 *μ*M) for 24 h and assayed for CYP1A1 enzyme activity by EROD assay, *n* = 4 ± s.d. (b) 5F 203 induces CYP1A1 and CYP1B1 mRNA levels in sensitive (MCF-7) cells. MCF-7 and AHR100 cells were treated with 5F 203 (1 *μ*M) for 24 h, RNA was isolated from control and treated samples, and CYP1A1 and CYP1B1 gene expression were measured by real-time RT-PCR as described. Data are shown as fold induction of treated cells relative to constitutive expression in control cells ± s.d., *n* = 7 (samples from two independent experiments) [17].

We also observed an increase in the transcription of AhR target genes such as CYP1A1/1B1, but AHR 100 cells, derived from MCF-7 which do not express the AhR receptor, were resistant to 5F 203, and the treatment did not induce activation of CYP1A1 (Figure 5) [17].

**Figure 6:**
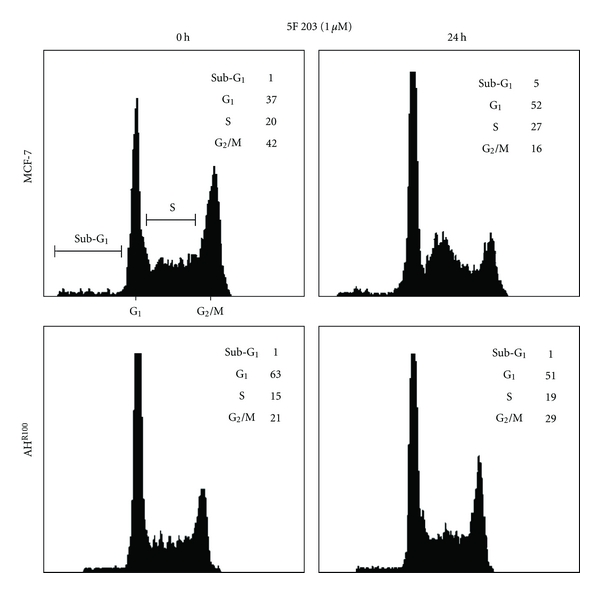
5F 203 induces some accumulation of cells in S phase. Exponentially growing cells (MCF-7 and AHR100) were exposed to either 0.1% DMSO (control) or 5F 203 (1 *μ*M) for 24 h, harvested, washed in PBS, and fixed in 70% ethanol. DNA was stained by incubating the cells in PBS containing propidium iodide, and fluorescence was measured and analysed [17].

We also demonstrated that in MCF-7 cells, treatment with 5F 203 prevents entry into G2/M and S phase and causes apoptosis, which was not observed in AHR 100 cells (Figure 6) [17]. These data suggested that activation of AhR was necessary for the antitumor activity of the benzothiazoles in MCF-7. 

**Figure 7:**
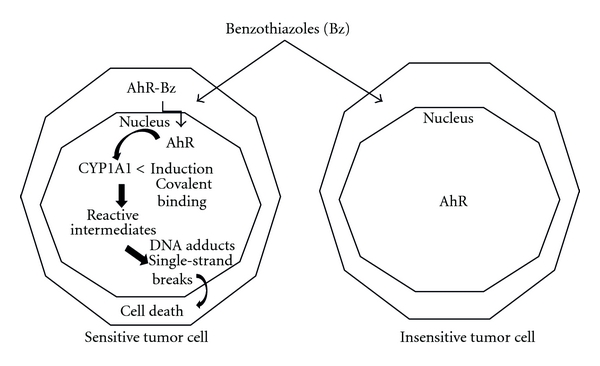
Mechanism of action of benzothiazoles.

The mechanism of action of benzothiazoles is represented in Figure 7.

## 6. Aminoflavone, an Alternative Therapy for ER+ Breast Cancer Cell Lines Resistant to Antihormone Treatment

**Figure 8:**
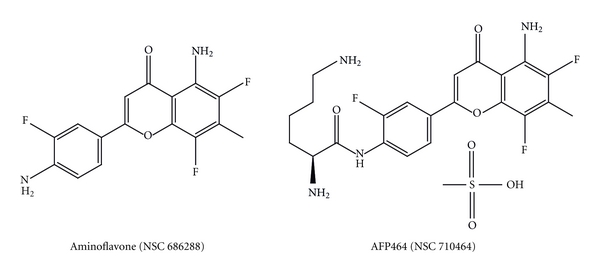
Aminoflavones' structures.

AF (NSC 686288) is a novel anticancer agent (Figure 8). Previous work from our research group demonstrated that AF is a ligand of AhR [19]. It was proposed that induction of CYP1A1 and high covalent binding of AF metabolites are markers to predict sensitivity to this drug in breast and renal tumors [19, 20]. AF derivative compound, aminoflavone prodrug (AFP464, NSC710464), has currently entered phase II clinical trials (Figure 8).

**Figure 9:**
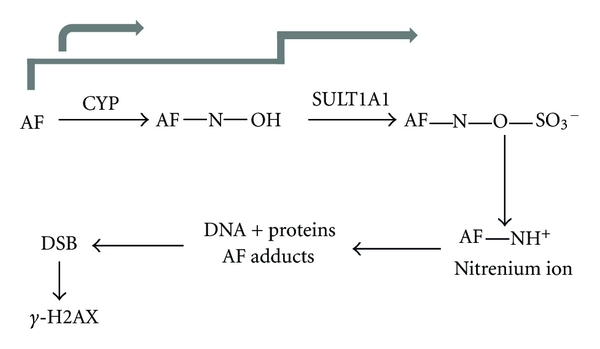
Mode of action of aminoflavone. Aminoflavone binds to cytosolic AhR. Only breast cancer cells with cytosolic AhR form AhR: AF complexes that translocate to the nucleus. Cells with constitutive nuclear AhR expression are not influenced by AF. In the nucleus, AhR: AF complex induces CYP1A1 leading to AF metabolism and DNA-damaging products. AF-sensitive breast cancer cells examined to this point express estrogen receptor (ER). SULT1A1: sulfotransferase 1A1; DSB: double strand breaks [30].

AF activity has been linked to the presence of cytoplasmic AhR and nuclear translocation of the AhR-AF complex followed by induction of cytochrome P450 (CYP) 1A1, activation of sulfotransferase 1A1 (SULT1A1), and DNA damage caused by metabolites. The latter is exemplified by the occurrence of gamma-histone 2AX (H2AX) phosphorylation consistent with induction of DNA single-strand breaks and DNA-protein cross-links (Figure 9) [30]. 

**Figure 10:**
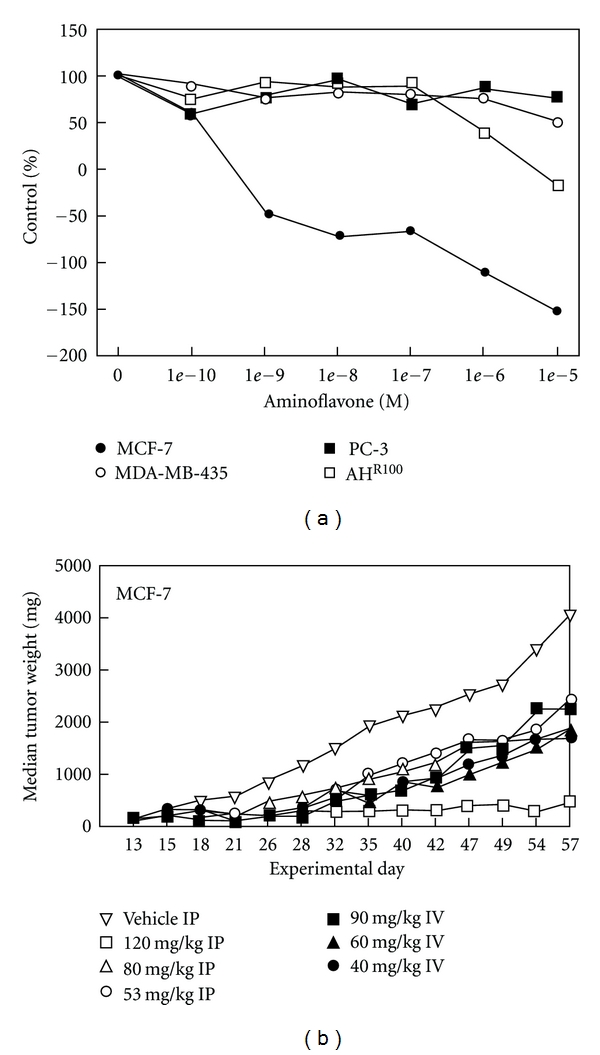
Cytotoxicity of aminoflavone *in vitro* and *in vivo*. (a) Selective cytotoxicity of aminoflavone to MCF-7 breast tumor cells. Cell lines were seeded into 24-well plates and allowed to grow for 48 hours. Cells were treated with 10^−10^ to 10^−5^ mol/L aminoflavone for an additional 72 hours. Cell monolayers were stained with sulforhodamine (b) and protein was determined spectrophotometrically. Points, mean ± SD (*n* = 10). SD was <5% for all drug concentrations and was omitted for purpose of graphical clarity. (b) Antitumor activity of aminoflavone against MCF-7 breast tumor xenografts. Treatments were given on a QD × 5 schedule beginning on day 13. There were 20 mice in the vehicle control group and 6 mice per dose of aminoflavone in the treated groups [19].

AF has shown exquisite *in vitro* sensitivity toward estrogen-receptor-positive (ER+) breast cancer cell lines and *in vivo* activity in MCF-7 xenografts (Figure 10) [19]. 

In contrast, ER− breast cancer cell lines, like MDA-MB-431, were resistant to AF. Crosstalk between AhR and ER signaling pathways has been established. It was shown that ligand-bound AhR can mimic estrogens and redirect ER from ER target genes to AhR target genes, such as CYP1A1 [13]. Given these facts, patients that might benefit most from AF treatment in the clinic could be those with ER+, endocrine-therapy-resistant breast cancers.

Recent work was performed to test whether breast cancers resistant to antihormone treatments retain sensitivity to AF. The AF response in a panel of molecularly well defined breast cancer cell lines was evaluated. The latter included MCF-7 (ER+) and its resistant subclones MCF-7/Her2-18 (ER+), MCF-7TAM1 (ER+), LTLC (ER+^high^), and LTLT (ER^very  low^); T47D (ER+), MDA-MB-231 (ER−); Hs5718ti8 (ER−); MCF10A (ER−). Antiproliferative effects were measured by MTT assay, and concentrations that inhibit cell growth by 50% (IC_50_) of the control were established for each cell line. The response to AF was compared to ER and AhR expression by western blot and immunocytochemistry. AF potently inhibited the growth of all ER+ breast cancer cell lines at nanomolar concentrations irrespective of hormone resistance (mean IC_50_s: MCF-7 = 16 nM; MCF-7/Her2-18 = 20 nM; MCF-7TAM1 = 25 nM; T47D = 14 nM; LTLC = 100 nM), whereas the ER− breast cancer cell lines (mean IC_50_s: MDA-MB-231 = 25 *μ*M; Hs5718ti8 = 18  *μ*M; LTLT >50 *μ*M) and the ER− breast epithelial line MCF10A (IC_50_ = 3 *μ*M) were 2- to 3-log-fold less sensitive. Interestingly, AhR was predominantly localized in the nuclei of all ER− cell lines, but was expressed in the cytoplasm of ER+ cells. MCF-7/Her2-18 and MCF-7TAM1, which are both tamoxifen-resistant subclones of MCF-7 (IC_50_s 4–OH–tamoxifen 3 and 10 *μ*M) and LTLC, a letrozole-resistant clone (IC_50_ > 1 *μ*M), retained a sensitivity to AF that was similar to parental MCF-7 cells [31, 32].

In order to examine the role of ER in AF sensitivity, AF was combined with a fixed concentration (100 nM) of the “pure” antiestrogen Faslodex in MCF-7 cells. The IC_50_ for AF plus Faslodex was found to be 0.5 nM, suggesting a synergism between the two drugs. To further prove that ER-AhR crosstalk is correlated with AF sensitivity, MDA-MB-231 cells (ER−) were stably transfected with human estrogen receptor-*α*, rendering them ER+. It was found that the ER+ MDA-MB-231 cells had cytoplasmic AhR and were 5 times more sensitive to AF (IC_50_ = 5 *μ*M) compared to parental- and vector-transfected cells. 

The authors concluded that the cytoplasmic AF-AhR complex can activate unliganded ER to enhance AhR target gene expression [31, 32].

## 7. Vorinostat Can Sensitize Triple Negative Breast Cancer Cell Lines to Aminoflavone Prodrug

AFP464 exhibits differential *in vitro* cytotoxicity in breast cancer cell lines with inhibitory 50% (IC_50_) concentrations ranging from 0.01 to 30 *μ*M. AFP464 plasma levels that can safely be reached in patients are *∼*1 *μ*M. In sensitive cells, AFP464 induces AhR-mediated cytochrome P450- (CYP-) dependent xenobiotic response and cell death. In resistant cells, the CYP system was not induced. Recent experiments showed that a panel of 10 luminal and basal A type breast cancer cells irrespective of resistance to antihormone therapies (e.g., tamoxifen refractory MCF7TAM1 cells) were exquisitely sensitive to AFP464 with IC_50_s between 0.01 and 0.025 *μ*M, whereas “triple-negative” breast cancer (TNBC) cell lines with a basal B-like gene cluster were resistant [33, 34]. Drug concentrations needed to inhibit the growth of basal B-like cells by 50% (25–30 *μ*M) may not be achieved in patients. Thus, it was proposed that in TNBCs, combination treatments will be needed and that agents modifying gene transcription, such as the histone deacetylase inhibitor, vorinostat, might be suitable combination partners. To test this hypothesis, combination experiments were employed using the fixed IC_50_ ratio method and treated MDA-MB-231 and Hs578T cells were treated for 24, 48, and 72 hrs with vorinostat followed by AFP464 for a total of 5 days. It was found that AFP464 and vorinostat can act synergistically; in Hs578T cells, combination indices (CIs) of <0.3 were seen after pretreatment with vorinostat for 24 hrs, reducing the AFP464 IC_50_ from 20 *μ*M to 0.5 *μ*M; in MDA-MB-231 cells, CIs indicating synergism (<1) were observed when adding AFP464 after 48 and 72 hrs pretreatment with vorinostat. This led to a 25-fold sensitization to AFP464 (IC_50_ = 1 *μ*M) [33, 34].

To study mechanisms that could explain the sensitization of TNBC cell lines to AFP464, real-time PCR to assess the induction of CYP1A1 and CYP1B1 after vorinostat treatment, western blotting to determine estrogen receptor reactivation, and transcriptional profiling using Illumina Human HT-12 v3 whole-genome expression BeadChips were performed. In MDA-MB-231 cells vorinostat treatment restored the AhR-dependent xenobiotic response to AFP464 by inducing both CYP1A1 and B1; also estrogen receptor expression was detectable in MDA-MB-231 and Hs578T cells at the protein level, consistent with driving these TNBCs into a more luminal-like genotype. These data indicated the usefulness of gene expression profiling in selecting patients for AFP464 treatment. While single agent therapy might present an option for hormone refractory luminal and basal A type patient populations, breast cancer patients with basal B-like tumors will require combination therapies, for example, with vorinostat. Pretreatment of TNBCs with vorinostat could sensitize these tumors to AFP464 [34].

## 8. Nuclear Expression of the Aryl Hydrocarbon Receptor Elicits Resistance to Aminoflavone Prodrug

Other studies were performed to test whether primary human tumors would also show nuclear or cytoplasmic AhR and to assess the extent to which AhR was expressed. 165 archival human tissues were analyzed comprising breast, pancreas, ovarian, and renal cell cancers. It was found that the 59 percent of all cases had detectable AhR, amongst those 78 percent exhibited cytoplasmic AhR and 22 percent nuclear AhR. Pancreatic (70%) and breast cancers (46%) showed the highest percentage of cytoplasmic AhR [34].

Together these data indicate that AhR has a distinct distribution pattern in tumor cells. Cytoplasmic AhR expression elicits sensitivity to the AhR ligand AFP464. If AhR is located in the nucleus, the xenobiotic response is impaired and AFP464 cannot be activated [35].

## 9. Conclusions

These results lead one to the conclusion that AF and Bzs, two structurally dissimilar compounds, share certain characteristics in their mechanism of action but are certainly not identical in their pattern of activity. Both compounds activate the AhR signaling pathway leading to an increase in CYP1A1 and CYP1B1 gene expression in drug-sensitive MCF-7 cells but not in resistant cells like MDA-MB-435, PC-3, or MCF-7 AhR-deficient AHR100 cells. Although activation of the AhR signaling pathway by the drugs may be necessary for increases in CYP1A1 and CYP1B1 gene expression, additional metabolic conversions may be necessary to produce cytotoxicity. These results suggest that the cytotoxicity of AF and Bz in a sensitive breast tumor cell line is the result of the engagement of AhR-mediated signal transduction [17, 19]. Thus, these drugs, unlike other neoplastic agents, require AhR-mediated signaling to cause DNA damage. This offers a new potential treatment strategy for breast cancers with intact AhR signaling. Induction of CYP1A1 and AhR activation were considered as markers to predict sensitivity of tumors to Bz and AF treatment in Phase I clinical trials.

The data presented in this paper indicate that the cytoplasmic AF-AhR complex can activate unliganded ER to enhance AhR target gene expression and as a result AF cytotoxicity [32]. Therefore, Phase II clinical trials of AF should include hormone-resistant, metastatic breast cancers. 

Also, these data indicated the usefulness of gene expression profiling in selecting patients for AF treatment. While single-agent therapy might present an option for hormone refractory luminal and basal A type patient populations, breast cancer patients with basal B-like tumors will require combination therapies, for example, with vorinostat [33, 34]. Pretreatment of TNBCs with vorinostat could sensitize these tumors to AF, and this should be exploited in clinical trials. 

AhR has a distinct distribution pattern in tumor cells. Cytoplasmic AhR expression elicits sensitivity to the AhR ligand AF. If AhR is located in the nucleus, xenobiotic response is impaired and AF cannot be activated [35]. Thus, immunohistological analysis of AhR should also be considered as a tool in the upcoming Phase II trials in breast cancer to select patients that are most likely to benefit from AF treatment.
